# Red Blood Cell Distribution Width-to-Platelet Count Ratio as a Prognostic Marker for Predicting Severity and Various Outcomes in Acute Pancreatitis

**DOI:** 10.7759/cureus.81747

**Published:** 2025-04-05

**Authors:** Sidharth Arora, Shubhransu Patro, Vibha Sharma, Arushi Choudhary, Shubham Desale, Preetam Nath

**Affiliations:** 1 Endocrinology, Sher-i-Kashmir Institute of Medical Sciences, Srinagar, IND; 2 General Medicine, Kalinga Institute of Medical Sciences, Kalinga Institute of Industrial Technology (KIIT) Deemed to be University, Bhubaneswar, IND; 3 Gastroenterology and Hepatology, Kalinga Institute of Medical Sciences, Kalinga Institute of Industrial Technology (KIIT) Deemed to be University, Bhubaneswar, IND

**Keywords:** acute pancreatitis, pancreatitis, pancreatitis complications, pancreatitis outcomes, rpr, severity of pancreatitis

## Abstract

Introduction: The natural course of acute pancreatitis is quite variable, where most patients with mild acute pancreatitis usually follow a self-limiting course, whereas the mortality is quite higher in severe acute pancreatitis. Hence, early prediction of severity is essential for proper triaging. The study aimed to assess the diagnostic accuracy of red blood cell distribution width (RDW)-to-platelet count ratio (RPR) as a prognostic marker in acute pancreatitis.

Methods: It was a prospective, observational study conducted on consecutive patients with acute pancreatitis. All participants were subjected to routine laboratory investigations and radiological evaluation using transabdominal ultrasound at the time of admission and a contrast-enhanced CT scan after 96 hours. All the parameters, including RPR, and various severity scores, such as the Systemic Inflammatory Response Syndrome (SIRS) score, Ranson score, Bedside Index for Severity in Acute Pancreatitis (BISAP) score, and Modified Marshall score, were compared between patients with mild or moderate to severe acute pancreatitis by using standard statistical tests.

Results: A total of 200 patients diagnosed with acute pancreatitis were enrolled in this study, out of which the most common etiologies of acute pancreatitis were alcohol abuse (88 (44%)) and gallstones (57 (28.5%)). The overall mortality in our patients was seven (3.5%), which occurred only in severe acute pancreatitis. The mean RPR of patients with mild and moderately severe to severe acute pancreatitis were 0.07 ± 0.02 and 0.12 ± 0.09, respectively (p < 0.05), whereas the mean RPR of patients who survived and those who did not were 0.09 ± 0.06 and 0.12 ± 0.05, respectively (p < 0.05).

Conclusion: RPR calculated at the time of admission is found to be an independent prognostic marker in acute pancreatitis with the potential to identify individuals at risk for developing severe acute pancreatitis as well as mortality.

## Introduction

Acute pancreatitis is one of the most common causes of hospitalization due to gastrointestinal diseases. The pathophysiology of acute pancreatitis is characterized by local as well as systemic inflammatory responses that may lead to multiorgan failure. Its natural course is quite variable, and in the majority of patients, it follows a mild and self-limiting course with a mortality rate of less than 1% [1]. However, a considerable proportion of patients (approximately 20%) may develop moderate to severe disease with intra- and/or extra-pancreatic necrosis, which may be associated with the presence of multiple organ failures, resulting in high mortality [2]. Mortality in the initial two weeks is usually due to multiorgan dysfunctions occurring as a result of systemic inflammatory response syndrome (SIRS), whereas mortality in the subsequent second phase is because of infective complications of localized collections [2].

The differentiation between mild to moderate and severe acute pancreatitis is based on clinical, laboratory, and radiological assessments which have been evaluated in several studies. Various single clinical or laboratory parameters (like SIRS, C-reactive protein (CRP), serum creatinine, blood sugar, and packed cell volume) as well as multi-parameter scores (such as the Ranson score, Glasgow score, and Acute Physiology and Chronic Health Evaluation II (APACHE II) score) have been studied to predict the severity as well as mortality in patients suffering from acute pancreatitis [3-5]. However, most of the above multi-parameter scores are cumbersome to calculate, which limits their utility in clinical practice. An ideal prognostic test/score for acute pancreatitis is the need of the hour, which should be readily available and can be calculated easily at the bedside. The complete/full blood count (CBC) is a simple blood test used everywhere, and it consists of various red blood cell (RBC)/erythrocyte, white blood cell (WBC)/leucocyte, and platelet counts with their indices. Red blood cell distribution width (RDW) is one of the important RBC indices, which measures the degree of variability in the volume and size of RBCs. Several studies have reported that RDW is an important prognostic marker for various diseases, such as celiac disease, colon cancer, and acute myocardial infarction [6-8]. In individuals with celiac disease, an elevated RDW has been suggested as a potential marker for severe intestinal atrophy [6]. Similarly, elevated RDW has also been linked to poor prognosis and risk for metastatic disease in colorectal cancer [7]. Furthermore, in acute myocardial infarction, studies have suggested elevated RDW to be associated with poor prognosis and increased mortality [8]. RDW has been shown to be increased in these acute as well as chronic diseases. In contrast, the platelet count usually decreases in several diseases, especially liver diseases. Hence, RDW and platelet count can serve as ideal biomarkers for the severity of these diseases, and the RDW-to-platelet count ratio (RPR) can be a more useful predictor. As acute pancreatitis is associated with SIRS in the early as well as late infective phase and the release of inflammatory cytokines, an increase in RDW occurs due to the release of premature RBCs into the circulation. Furthermore, the platelet count usually decreases whenever SIRS becomes more severe, which may be due to the bone marrow suppressive effect of pro-inflammatory cytokines and consumption due to disseminated intravascular coagulation (DIC). Recently, RPR has been demonstrated to be a prognostic marker of several medical conditions such as primary biliary cholangitis, liver fibrosis, liver cirrhosis, and acute myocardial infarction [9-11]. Furthermore, several retrospective studies have also shown the promising role of RPR as one of the prognostic markers in acute pancreatitis [12,13]. 

Study objectives

This study aims to evaluate the diagnostic accuracy of RPR in predicting the severity and outcomes of acute pancreatitis, including (1) assessing RPR as a prognostic marker for predicting organ failure, length of intensive care unit (ICU) stay, and mortality in acute pancreatitis and (2) comparing RPR with existing prognostic scores, such as the Bedside Index for Severity in Acute Pancreatitis (BISAP) score, Sequential Organ Failure Assessment (SOFA) score, and Modified Marshall score, to determine its relative effectiveness.

## Materials and methods

Study design and setting

This was a hospital-based prospective, observational study conducted at Kalinga Institute of Medical Sciences from September 2019 to August 2020.

Patient selection

Patients admitted with a confirmed diagnosis of acute pancreatitis were screened for eligibility. The inclusion criteria were as follows: (1) patients aged >18 years and (2) those with a diagnosis of acute pancreatitis based on the Revised Atlanta Criteria [14] and suffering from any two of the three criteria: (a) pancreatic-type abdominal pain, (b) elevated serum amylase and/or lipase more than three times the upper normal reference limit, and (c) imaging findings (CT or MRI) consistent with acute pancreatitis. 

In contrast, the exclusion criteria were as follows: (1) patients with a history of any form of anemia (anemia due to iron deficiency, megaloblastic anemia, or anemia of chronic disease) and hematological disorders (thalassemia or hemolytic disease), (2) patients with active malignant disease, (3) patients with inflammatory bowel disease, and (4) pregnant or lactating women.

Data collection and measurements

After enrolment, a detailed clinical history was collected and a physical examination was performed. All participants were subjected to appropriate laboratory investigations, including CBC, serum electrolytes, serum amylase, serum lipase, serum liver and renal function tests, serum CRP, lactate dehydrogenase (LDH), procalcitonin, and blood gas analysis. Radiological evaluation was performed using transabdominal ultrasound at the time of admission, and an abdominal contrast-enhanced CT scan was performed 96 hours after the onset of abdominal pain. 

Demographic and clinical data, such as time from date of pain onset to date of admission, etiology, comorbid diseases, pancreatitis subtypes (interstitial or necrotizing), severity of the disease, length/duration of hospital stay, as well as intensive care unit (ICU) stay, in-hospital mortality, need for vasopressor drugs, and mechanical ventilator requirements, were noted. SIRS was defined as the presence of two or more of the four criteria, such as increased heart rate exceeding 90 beats per minute, increased respiratory rate of more than 20 cycles per minute, abnormal body temperature (>38°C or <36°C), and abnormal total leucocyte count (>12,000/mm^3^ or 4000/mm^3^). The etiological diagnosis was attempted for each case. 

Study variables and prognostic scoring

RPR was calculated using the following formula: RPR = RDW(%)/platelet (10^9^/L). Various severity scores were recorded in the first 24 hours, such as the SIRS score, Ranson score, BISAP score, SOFA score, and Modified Marshall score. All patients were classified into three groups, mild, moderately severe, and severe pancreatitis, according to the Revised Atlanta Classification [14] (Table 1). However, for statistical analysis, patients were divided into two groups: the mild acute pancreatitis group and the severe acute pancreatitis group (which includes both moderate and severe acute pancreatitis). All participants were followed up till death or three months after admission, whichever occurred earlier. The various outcomes of acute pancreatitis were assessed, such as organ failure, duration/length of hospital stay, duration/length of ICU stay, and mortality.

**Table 1:** Revised Atlanta Classification for severity of acute pancreatitis

Severity of acute pancreatitis	Local complications	Organ failures
Mild	Absent	Absent
Moderate	Present	Transient
Severe	Present/absent	Persistent

Statistical analysis

All numerical data were expressed as means and standard deviations, whereas categorical data were presented as frequencies and percentage tables. Numerical variables were compared by using Student's independent t-test, while the chi-squared test was used for categorical variables. The data were initially collected in Microsoft Excel 2017 (Microsoft Corp., Redmond, WA, USA) and analyzed with IBM SPSS Statistics for Windows, V. 20.0 (IBM Corp., Armonk, NY, USA). The normality of the data was assessed by the Kolmogorov-Smirnov test. The normally distributed (parametric) data were expressed as means with standard deviations, whereas data without normal distribution were shown as medians with inter-quartile ranges. Receiver operating characteristic (ROC) curve analysis was performed to study the predictors for primary as well as secondary outcomes. A p-value of <0.05 is considered significant.

Ethical considerations

The study adhered to the Declaration of Helsinki guidelines, and all patient data were anonymized prior to analysis. This study was approved by the Institutional Ethics Committee of Kalinga Institute of Medical Sciences (approval number: KIMS/R&D/162/2018), and written informed consent was collected from each study participant before enrolment.

## Results

A total of 268 eligible patients with acute pancreatitis were screened, out of which 200 patients were enrolled in the present study. The majority of patients (163 (81.5%)) were males. The mean age of all patients was 40.3 years (standard deviation was 14.4 years). One hundred twenty-one (60.5%) patients had mild acute pancreatitis, whereas the rest (79 (39.5%)) had moderate to severe acute pancreatitis. The most common etiology of acute pancreatitis was alcohol abuse (88 (44%)) followed by gallstones (57 (28.5%)), hypertriglyceridemia (12 (6%)), and other causes (Table 2). The etiology of acute pancreatitis remained unknown in 28 (14%) patients. A baseline comparison of all the characteristics between mild and moderate to severe pancreatitis has been depicted in Table 3. Patients with moderate to severe pancreatitis were older and had a higher incidence of SIRS, significantly higher values of RPR score, BISAP score, SOFA score, and Modified Marshall score, and longer length of hospital stay and ICU stay than those with mild acute pancreatitis. Out of all the patients, only seven succumbed to their illness and had belonged to severe acute pancreatitis. A comparison of all baseline parameters was also done for the survivors and those who did not survive (non-survivors) (Table 4). Patients who died had a significantly higher incidence of SIRS, longer duration of hospital as well as ICU stay, more number of organ failures, and higher mean RPR scores, BISAP scores, SOFA scores, and Modified Marshall scores than those who survived. 

**Table 2:** Etiology of acute pancreatitis in all patients DKA: diabetic ketoacidosis; ERCP: endoscopic retrograde cholangiopancreatography

Etiology	N	%
Alcohol abuse	88	44%
Gallstones	57	28.5%
Hypertriglyceridemia	12	6%
Steroid	4	2%
Ampullary stenosis	2	1%
Ascariasis	2	1%
DKA	2	1%
Hypercalcemia	2	1%
Pancreatic divisum	2	1%
Post-ERCP	1	0.5%
Unknown	28	14%

**Table 3:** Baseline characteristics of patients with mild and moderate to severe AP AP: acute pancreatitis; SD: standard deviation; SIRS: systemic inflammatory response syndrome; ICU: intensive care unit; RPR: red blood cell distribution width-to-platelet count ratio; BISAP: Bedside Index for Severity in Acute Pancreatitis; SOFA: Sequential Organ Failure Assessment

Parameters	Mild AP (n = 121)	Moderate to severe AP (n = 79)	P-value
Age (years) (mean ± SD)	39.66 ± 14.62	41.19 ± 14.40	>0.05
Male (n (%))	94 (77.68%)	69 (87.34%)	>0.05
Presence of SIRS (n (%))	25 (20.66%)	53 (67.09%)	<0.05
Duration of hospital stay (days) (mean ± SD)	7.11 ± 4.03	14.14 ± 10.40	<0.05
Duration of ICU stay (days) (mean ± SD)	4.00 ± 0.00	10.87 ± 9.54	<0.05
Number of organ failure(s) (n (%))			
No organ failure	121 (100%)	24 (30.38%)	<0.05
1	0	37 (46.84%)	
2	0	15 (18.99%)	
3 or more	0	3 (3.79%)	
Prognostic scores			
RPR (mean ± SD)	0.07 ± 0.02	0.12 ± 0.09	<0.05
BISAP (mean ± SD)	0.41 ± 0.68	1.95 ± 1.14	<0.05
SOFA (mean ± SD)	0.39 ± 0.81	3.00 ± 2.45	<0.05
Modified Marshall (mean ± SD)	0.00 ± 0.00	1.78 ± 1.68	<0.05
Mortality (n (%))	0	7 (8.86%)	<0.05

**Table 4:** Baseline characteristics of survivors and non-survivors AP: acute pancreatitis; SD: standard deviation; SIRS: systemic inflammatory response syndrome; ICU: intensive care unit; RPR: red blood cell distribution width-to-platelet count ratio; BISAP: Bedside Index for Severity in Acute Pancreatitis; SOFA: Sequential Organ Failure Assessment

Parameters	Survivors (n = 193)	Non-survivors (n = 7)	P-value
Age (years) (mean ± SD)	40.44 ± 14.60	38.57 ± 11.77	>0.05
Male (n (%))	158 (81.86%)	5 (71.43%)	>0.05
Presence of SIRS (n (%))	71 (36.79%)	7 (100%)	<0.05
Severity of AP			
Mild AP (n (%))	121 (62.69%)	0	<0.05
Severe AP (n (%))	72 (37.31%)	7 (100%)	
Duration of hospital stay (mean ± SD)	9.58 ± 7.74	18.43 ± 10.89	<0.05
Duration of ICU stay (mean ± SD)	9.00 ± 8.42	17.71 ± 11.16	<0.05
Number of organ failure			
No organ failure (n (%))	145 (75.13%)	0	<0.05
1 (n (%))	33 (17.09%)	4 (57.14%)	
2 (n (%))	12 (6.22%)	3 (42.86%)	
3 (n (%))	3 (1.55%)	0	
Prognostic scores			
RPR (mean ± SD)	0.09 ± 0.06	0.12 ± 0.05	<0.05
BISAP (mean ± SD)	0.96 ± 1.14	2.57 ± 0.53	<0.05
SOFA (mean ± SD)	1.30 ± 1.98	4.86 ± 2.54	<0.05
Modified Marshall (mean ± SD)	0.61 ± 1.22	3.43 ± 2.23	<0.05

**Figure 1:**
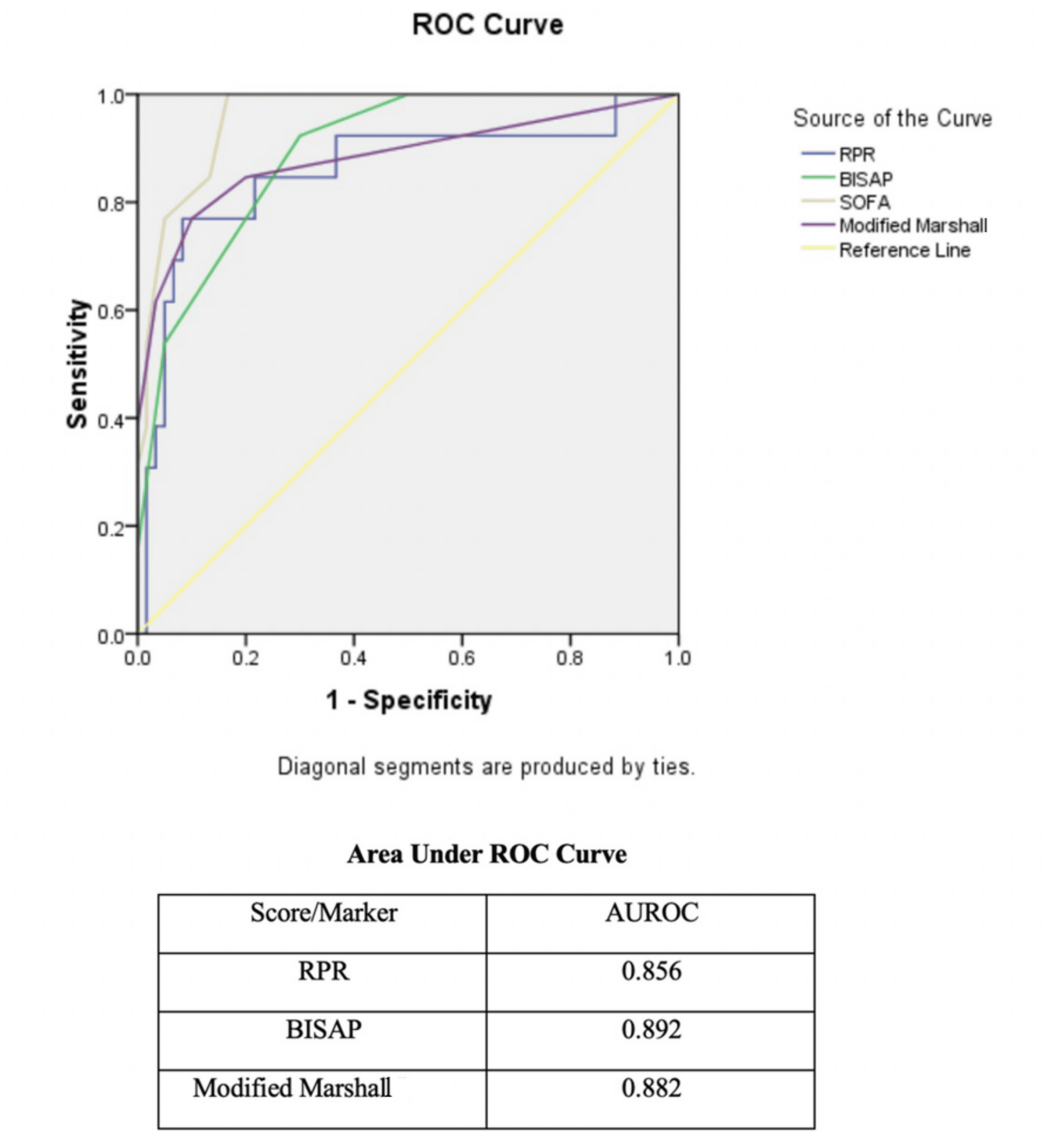
ROC curve for RPR, BISAP, and Modified Marshall scores ROC: receiver operating characteristic; RPR: red blood cell distribution width-to-platelet count ratio; BISAP: Bedside Index for Severity in Acute Pancreatitis; SOFA: Sequential Organ Failure Assessment; AUROC: area under the ROC curve

An ROC curve analysis was done, and above the cut-off value of 0.043, RPR had a sensitivity of 89.17% and specificity of 76.33% for the prediction of severe acute pancreatitis, whereas an RPR value above 0.067 had a sensitivity of 81.33% and specificity of 94.17% in predicting mortality in these patients with acute pancreatitis. The area under the ROC curve (AUROC) for RPR was 0.856, which was comparable with the AUROCs of the BISAP score (0.892) and the Modified Marshall score (0.882) (Figure 1).

## Discussion

In this prospective observational study, the majority (121 (60.5%)) had mild acute pancreatitis. The most common etiologies of acute pancreatitis were alcohol abuse followed by gallstones. In the present study, the mean age of the patients was 40.32 ± 14.43 years. One hundred sixty-three (81.5%) patients were males, which is similar to previous studies such as Çetinkaya et al. [12] and Barad et al. [13]. The overall mortality in our patients was 3.5%, which occurred only in severe acute pancreatitis. In the present study, the duration of ICU stay was <7 days in 19 (47.5%) patients, while it was 7-14 days and >14 days in 10 (25%) and 11 (27.5%) patients, respectively. The mean duration of ICU stay was 4.00 ± 0.00 days in patients who had mild acute pancreatitis, whereas it was 10.87 ± 9.54 days in moderately severe to severe acute pancreatitis (p < 0.05). These findings are in concordance with the study by Wu et al. [4].

The mean RPR of patients with mild and moderate to severe acute pancreatitis were 0.07 ± 0.02 and 0.12 ± 0.09, respectively (p < 0.05), in this study. These findings are similar to the study by Barad et al., where the mean RPR of patients with mild acute pancreatitis (0.038) was significantly lower than moderate to severe acute pancreatitis (0.0068) [13]. In the present study, the mean RPR of patients who survived and who did not were 0.09 ± 0.06 and 0.12 ± 0.05, respectively (p < 0.05). Çetinkaya et al. also observed higher median RPR in the non-survivors than the survivors in patients with acute pancreatitis [12]. Likewise, in the study by Barad et al., the mean RPR was significantly higher in patients with acute pancreatitis who expired (0.089) than those who were discharged (0.044) [13].

In the present study, the mean RDW and platelet values of patients were 14.62 ± 1.49% and 210.36 ± 90.63 × 10^9^/L, respectively. The length of hospital stay was <7 days in 73 (36.5%) patients, while it was 7-14 days in 93 (46.5%) and >14 days in 34 (17%) subjects, respectively. The mean length of hospital stay was 9.89 ± 8.00 days. Forty (20%) patients required ICU admission in our study.

In the present study, in ROC curve analysis, it was found that at a cut-off value of 0.043, RPR has a sensitivity of around 89.17% and specificity of around 76.33% in predicting the severity of the disease. This is similar to the study by Barad et al., where above 0.045, RPR had a sensitivity of 90% and specificity of 73% for predicting the disease severity [13]. In our study, ROC curve analysis revealed that above the cut-off value of 0.067, RPR had a sensitivity of 81.33% and specificity of 94.17% in predicting mortality in patients with acute pancreatitis. This is comparable to the studies of Barad et al. and Çetinkaya et al. [12,13]. The study by Çetinkaya et al. revealed that above the cut-off value of 0.000067, RPR could predict mortality with a sensitivity of 80.00% and a specificity of 70.08% with an AUROC of 0.783 (95% CI: 0.688-0.878) [12].

Strengths and limitations

The strengths of this study were its prospective design and the inclusion of all consecutive patients with acute pancreatitis. 

While our study provides valuable insights into the prognostic role of the RPR in acute pancreatitis, it is not without its limitations. Firstly, this study is a single-center retrospective analysis, which may limit the generalizability of our findings to broader populations or different healthcare settings. A multicenter study with a larger and more diverse cohort would strengthen the external validity of our results.

Secondly, although we included a reasonable sample size, the retrospective nature of the study introduces potential biases, including selection bias and information bias. Patient data were extracted from medical records, which may not always capture all relevant clinical details with uniform accuracy.

Thirdly, while we adjusted for key confounding variables in our analysis, unmeasured confounders could still influence the observed associations between RPR and disease severity. Future prospective studies with a more comprehensive data collection approach are needed to confirm our findings.

Additionally, we did not explore the dynamic changes in RPR over time. Serial measurements of RPR at different time points during hospitalization might provide a more robust understanding of its prognostic utility. Finally, although we compared RPR with established prognostic scores, further studies are necessary to assess its incremental value over existing tools and to determine optimal threshold values for clinical application.

Our study findings suggest the importance of RPR as a predictor for severity as well as mortality for patients with acute pancreatitis. Early identification of disease severity can help in implementing early therapeutic interventions which may lead to the reduction of morbidity and mortality. Severe acute pancreatitis will need careful monitoring as well as aggressive management preferably in an ICU for appropriate oxygen delivery and the maintenance of hemodynamics for better tissue perfusion. CBC is one of the simple, inexpensive, and easily available laboratory tests which is used routinely in our clinical practice. Indices of CBC such as RPR could be a useful and important marker for predicting the mortality of patients with acute pancreatitis.

## Conclusions

This study suggests RPR at the time of admission as an easy and promising prognostic marker for acute pancreatitis. Furthermore, RPR in acute pancreatitis has the potential to identify patients who are at risk for developing severe acute pancreatitis and improve their outcomes. However, prospective studies with validation in diverse patient populations are needed to establish the generalizability of these results.
